# Multipole Resonance in Arrays of Diamond Dielectric: A Metamaterial Perfect Absorber in the Visible Regime

**DOI:** 10.3390/nano9091222

**Published:** 2019-08-29

**Authors:** Chenhui Li, Haihua Fan, Qiaofeng Dai, Zhongchao Wei, Sheng Lan, Haiying Liu

**Affiliations:** Guangdong Provincial Key Laboratory of Nanophotonic Functional Materials and Devices, School of Information and Optoelectronic Science and Engineering, South China Normal University, Guangzhou 510006, China

**Keywords:** metamaterial, broadband perfect absorber, multipole resonance, subwavelength

## Abstract

Excellent characteristics and promising application prospects promote the rapid development of metamaterials. We have numerically proposed and demonstrated a novel subwavelength broadband metamaterial perfect absorber (BMPA) based on diamond dielectric arrays. The proposed absorber is composed of an ultra-thin two-layer structure covering the dielectric periodic array on a metal substrate. The materials of dielectric silicon (Si) and gold (Au) substrate are discussed in detail. In addition, different dielectric and refractory materials are also applied to achieve broadband absorption, which will make the proposed absorber greatly broaden the application field. A perfect absorption window (i.e., absorption rate exceeding 90%) can be obtained from near-ultraviolet to the visible range. The average absorption rate of 93.3% is achieved in the visible range. The results of multipole decomposition show that broadband absorption is mainly caused by electromagnetic dipole resonance and lattice resonance in a periodic array of Si. The proposed absorber can be extended freely by adjusting the structural parameters. The polarization-independent and incident angle insensitivity are proved. The proposed absorber may well be used in light energy acquisition, as well as for the scalability of optoelectronic and sensing devices.

## 1. Introduction

Metamaterials with extraordinary characteristics, which have attracted considerable attention and made significant progress over the past decades, are widely used in many fields [1,2,3,4,5,6]. Among them, the metamaterial perfect absorbers (MPAs) play an important role. MPAs have led to further development in fields such as thermal radiation [7,8], photoelectric detection [9,10], biosensor [11,12,13], energy-harvesting devices [14,15,16] and photo-thermal [17,18]. The metamaterial perfect absorber (MPA) based on metal-insulator-metal was proposed by Landy for the first time in 2008 [19]. Since then, various types of perfect absorbers have been achieved through experiments or simulations [20,21,22]. Most existing MPAs employ the noble metal with inherent absorption loss. The perfect absorption of the metal resonator is due to the existence of electric dipole resonance and surface plasmon resonance (SPR). For instance, rainbow trap operations have been demonstrated over a wide wavelength range in the multi-layer metamaterial structure [23]. However, Joule heat [24] produces unfavorable condition for noble metal resonators, which is fatal to devices. In order to solve the above problem, there are several ways to solve the high temperature constraint [25,26], and the use of refractories is one of them. Refractories such as titanium (Ti), titanium nitride (TiN), tungsten (W), vanadium (V) are selected as appropriate materials. Liu has used different refractories to implement nanoscale solar absorbers [26,27]. It is a pity that most of refractory materials cannot provide compatibility with complementary metal oxide semiconductor (CMOS) technology. Fortunately, optical Mie resonance has been observed in high-index dielectrics [28,29]. The method of multipole decomposition is used to evaluate the main contributions in dielectrics. In addition to the electric dipole resonance, the magnetic dipole resonance is also observed in the dielectrics. The loss of magnetic dipole resonance is smaller than that of electric dipole resonance, which makes it possible to realize the dielectric-based broadband metamaterial perfect absorber (DBMPA). High-index dielectrics such as silicon (Si), indium arsenide (InAs), indium phosphide (InP), germanium (Ge), gallium arsenide (GaAs) are selected as the functional layer to achieve electromagnetic resonance. As the preferred material of microelectronics, Si is very important to modern nanotechnology and has a bright future in the development of energy. Zhu et al. have implemented MPA based on Si nanostructure [30] and Liu et al. have proved semiconductor metasurface perfect absorber [31]. Of note, the bandwidth of the existing dielectric-based MPAs is insufficient and the structures are complex in the field of visible light. A DBMPA with suitable structural size, especially the subwavelength structure and capable of covering visible range would be highly desirable.

Inspired by earlier works, we have proposed and demonstrated a novel method of DBMPA. The materials of arrays of patterned Si and gold (Au) substrate are discussed in detail. A perfect absorption window was shown within 262–709 nm, where the average absorption rate was 95.5%. Analysis by multipole decomposition of diamond dielectric Si with electric dipole and magnetic dipole resonance coupling, the excitation of surface plasmon resonance of Au substrate and its coupling with dipole resonance, and lattice resonance are the main contributions to absorption. In addition, different dielectrics and refractories are applied to achieve broadband absorption. The excellent subwavelength structural properties and strong electromagnetic resonance of dielectric have important applications in the fields of thermal radiation, photoelectric detection, and solar energy.

## 2. Materials and Methods

**Figure 1:**
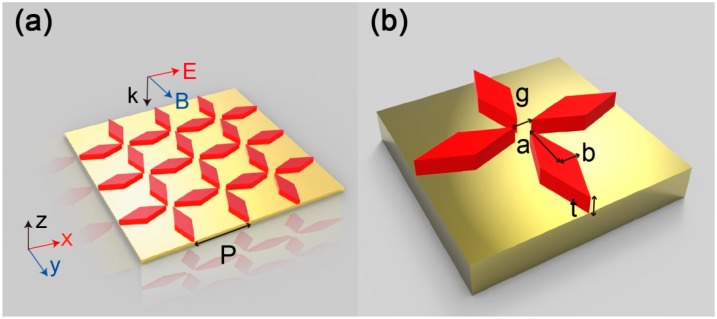
(**a**) Perspective views of the DBMPA, the upper red for Si, the lower for Au; (**b**) a unit cell, in which the thickness of the Au film is 100 nm, the thickness (t) of the Si resonator is 70 nm, the period (P) is 240 nm, the resonator is diamond, the semi-major axis (a) is 110 nm, the semi-minor axis (b) is 40 nm, and the gap (g) between the adjacent resonators is 20 nm. The tunable DBMPA can be further obtained by changing the structural parameters in the figure.

Firstly, we selected commonly used materials for detailed discussion, and then observed the absorption properties of other dielectric and refractory materials. A schematic diagram of the proposed DBMPA is shown in Figure 1a and that of the unit cell, 1b. The DBMPA has a two-layer subwavelength structure consisting of Au film and upper arrays of patterned Si. The Au, which is 100 nm, is mainly used to prevent light transmission and produce surface plasmon resonance. Electromagnetic resonance is provided on the resonators, and the unique structure allows the electric and magnetic dipole resonance to be concentrated in the same wavelength range, and the tip of the resonator can effectively bind the electric field. The thickness (t) of the Si resonator is 70 nm, the period (P) is 240 nm, the resonator is diamond, the semi-major axis (a) is 110 nm, the semi-minor axis (b) is 40 nm, and the gap (g) between the adjacent resonators is 20 nm. A three-dimensional finite-difference time-domain method (FDTD) is used to calculate the characteristics of the optical field and the resonance field. The periodic boundary conditions were defined in the x- and y-directions to reproduce this arrangement and save memory, and the z-direction uses a perfectly matched layer (PML) to eliminate scattering. In order to make the calculation results more accurate, a higher mesh order is used in the simulation process to refine the observed phenomenon, with the mesh size being λ/28, λ is the minimum wavelength of the simulation. The simulated light source is a planar wide-spectrum light source from 200 nm to 800 nm. The directions of the electric field and the magnetic field of the light source are parallel to the x and the y-direction, respectively, and the wave vector k is in the negative direction along the z-direction. The permittivity data of Si are obtained from the experiment of Palik, and Au is from Johnson and Christy [32]. Absorption is defined as A = 1 − R − T, where R and T are the reflectivity and transmittance, respectively, using a frequency-domain field and power monitor to record in three-dimensional simulation. In order to obtain perfect absorption, it is necessary to reduce reflection and transmission. Since the thickness of the Au film is greater than the penetration depth of electromagnetic waves in the near-ultraviolet to near-infrared region, the transmission is almost 0, that is, T = 0. The results of placing the monitor on the bottom of the metal indicate that the transmittance is less than 10^−5^ from 200 nm to 800 nm. Therefore, the absorption can be simplified to A = 1 − R, and only the reflectivity of the absorber needs to be measured. The preparation process of the metamaterial can adopt conventional nanotechnology such as deposition technique [33,34], and nanoimprint lithography [35,36].

## 3. Results and Discussion

**Figure 2:**
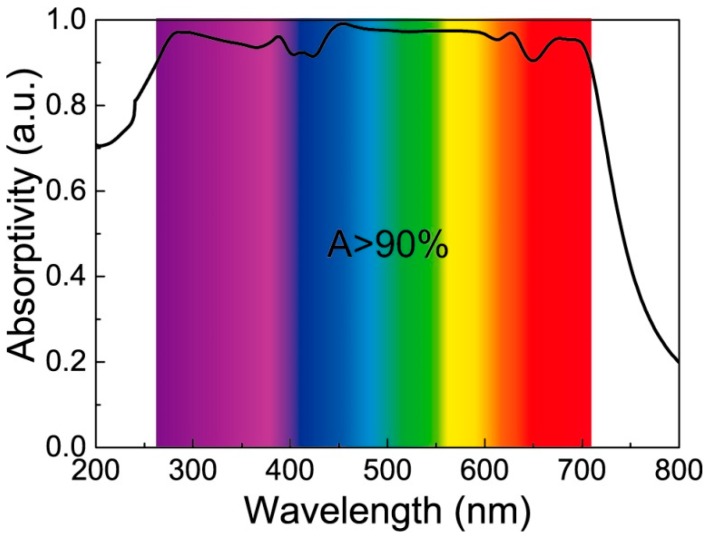
Simulation results of the proposed DBMPA, the black solid line represents the absorptivity from 200 nm to 800 nm, and the rainbow indicates that the absorption is greater than 90% of the wavelength corresponding to the wavelength color from 262 nm to 709 nm.

The structure shown in Figure 1 is simulated and numerically processed. In the absorption spectrum shown in Figure 2, the black solid line represents absorptivity. It can be seen that the perfect absorption window covers almost the entire visible range, including a part of the ultraviolet, and the maximum absorption is 99.1% at 453 nm. Absorption of more than 90% from 262 nm to 709 nm is expressed by the color corresponding to the wavelength. The absorption efficiency for the visible light range of 380–760 nm is calculated to be 93.3%. For the entire simulation range, the average absorption efficiency is 87.3%. The proposed DBMPA has remarkable absorption performance and noticeable potential applications from the above data.

**Figure 3:**
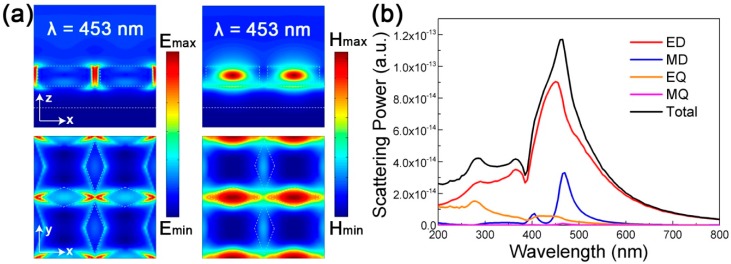
(**a**) The electric and magnetic field distributions for the proposed DBMPA at the absorption peak of 453 nm along the xoy and xoz plane; (**b**) scattering power of the multipoles as a function of the wavelength, where the resonator with semi-major axis is 110 nm and semi-minor axis is 40 nm, the contributions of the electric dipole (ED), magnetic dipole (MD), electric quadrupole (EQ), and magnetic quadrupole (MQ) to the total scattering are revealed.

To further understand the absorption response of the proposed DBMPA at different wavelengths, four wavelengths: λ_1_ = 368 nm, λ_2_ = 453 nm, λ_3_ = 627 nm and λ_4_ = 700 nm are selected. The electric and magnetic field intensity distribution at 453 nm (maximum absorption point) are shown in Figure 3a. The xoy and xoz planes are plotted. In Figure 3a, the electric field is mainly distributed between two adjacent resonators, while the strong magnetic field is distributed in the resonator and has a peak intensity point in the resonator, which proves that there is both electric and magnetic dipole resonance at this wavelength [37,38]. To further understand the contribution of electromagnetism, multipole decomposition of a single resonator is performed in free space, as shown in Figure 3b. According to the electromagnetic multipole decomposition theory of optical nanomaterials, the multipole decomposition diagram can be obtained according to the following formula [39,40,41].

(1) $$I=\frac{2\omega^{4}}{3c^{3}}{\left|p\right|}^{2}+\frac{2\omega^{4}}{3c^{3}}{\left|m\right|}^{2}+\frac{\omega^{6}}{5c^{5}}{\sum \left|Q_{\alpha \beta }\right|}^{2}+\frac{\omega^{6}}{20c^{5}}{\sum \left|M_{\alpha \beta }\right|}^{2},$$

(2) $$p=-\frac{1}{i\omega }\int jd^{3}r,$$

(3) $$m=\int \left(r\times j\right)d^{3}r,$$

(4) $$Q_{\alpha \beta }=\int \left[r_{\alpha }j_{\beta }+r_{\beta }j_{\alpha }-\frac{2}{3}(r\cdot j)\delta_{\alpha \beta }\right]d^{3}r,$$

(5) $$M_{\alpha \beta }=\frac{1}{3c}\int \left[{\left(r\times j\right)}_{\alpha }r_{\beta }+{\left(r\times j\right)}_{\beta }r_{\alpha }\right]d^{3}r.$$

where *ω* is the frequency of light, *c* is the speed of light in vacuum, *j* is the induced volume current and *α*, *β* are Cartesian components in the coordinate system. p, m, Q_αβ_, M_αβ_ are electric dipole moment, magnetic dipole moment, electric quadrupole tensors, magnetic quadrupole tensors, respectively.

Figure 3b clearly shows that the absorption is mainly due to the ED (electric dipole) and EQ (quadrupole) resonance, while the MD (magnetic dipole) and MQ (magnetic quadrupole) are negligible from 200 nm to 400 nm. The ED decreases from 600 nm to 700 nm. Absorption near 450 nm is mainly the result of the coupling of electric dipole resonance and magnetic dipole resonance, and the maximum absorption at 453 nm is 99.1%. The coupling effect is obtained by optimizing structural parameters of the Si resonator, and these parameters such as thickness, semi-major axis and semi-minor axis, will affect the position and strength of the resonance peak. The electromagnetic field intensity distribution of Figure 3a is in good agreement with the multipole decomposition of Figure 3b.

**Figure 4:**
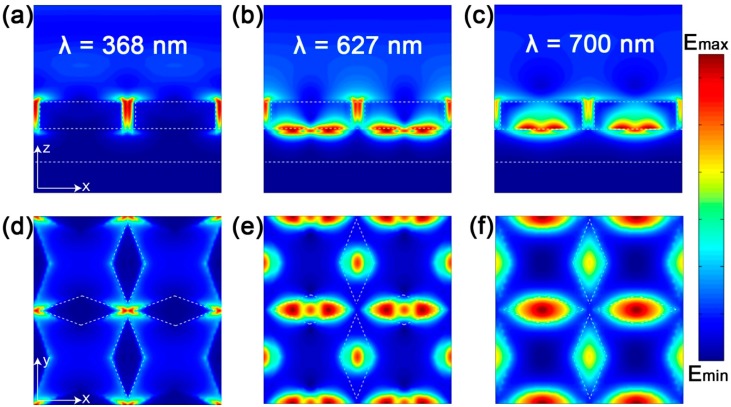
Corresponding electric field distribution at 368 nm, 627 nm and 700 nm, where (**a**–**c**) is the electric field intensity distribution in the xoy plane, and (**d**–**f**) is the electric field intensity distribution in the xoz plane.

The electric field distribution map of 368 nm, 627 nm and 700 nm in the xoy plane (a–c) and xoz plane (d–f), is shown in Figure 4. For the wavelength of 368 nm, as shown in Figure 4a,d, the strong electric field is mainly distributed between two adjacent resonators, and it can be obtained by multipole decomposition where the absorption is the result of the electric dipole resonance excitation of the resonator. In multipole decomposition, the MD, EQ, MQ are negligible from 600 nm to 800 nm, and the electric field distribution at 627 nm is concentrated mainly between the bottom of the resonator and gold film. Resonators, which are parallel to the electric field, are strongly coupled to incident light, and others help in absorption. There is weak electric field distribution between the resonators (such as Figure 4b,e), and two peak points were observed at the resonators, which confirm that the absorption at 627 nm is the main result of the dipole resonance of the resonators and its coupling with the SPR of substrate. Figure 4c,f show that the electric field is mainly concentrated in the resonator, and there is a maximum intensity on the resonator parallel to the electric field direction. This result indicates that lattice resonance is the main reason for absorption at 700 nm, and the electric field is confined to the resonator due to the excitation of lattice resonance [42,43].

**Figure 5:**
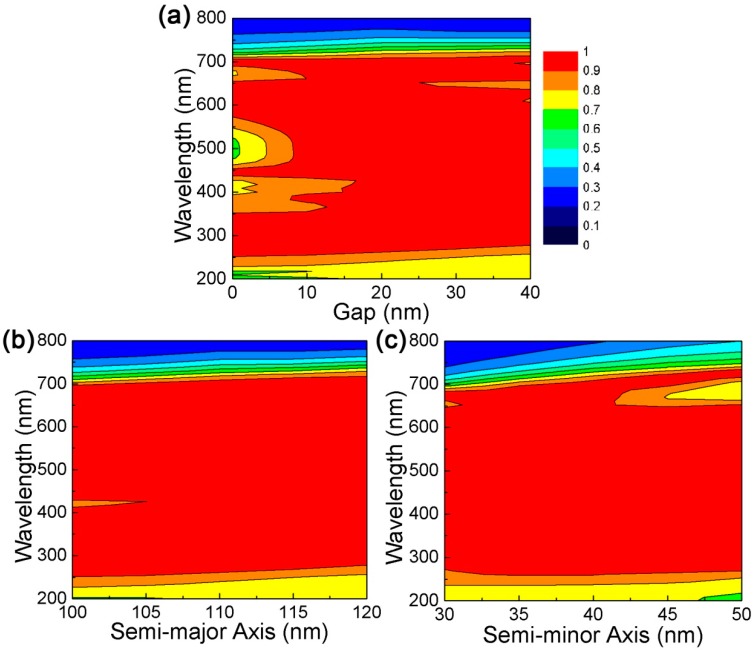
Tunable DBMPA is obtained by changing the structure parameters. (**a**) the gap increases from 0 to 40 nm; (**b**) the change of the absorption spectrum caused by the change of semi-major axis (a) of the resonator; (**c**) the change of the absorption spectrum caused by the change of semi-minor axis (b) of the resonator from 30 to 50 nm.

In order to further understand the working mechanism of the proposed DBMPA, the absorption response under different structural parameters has been studied. Figure 5a illustrates the response of the absorber when the gap (g) between adjacent resonators is changed. With the increase of parameter g, the spectral curve has only a slight red shift in the wavelength range. Two main characteristics were observed: the absorption rate continued to increase in the shorter wavelength range, and the absorption bandwidth and absorptivity decreased in the longer wavelength range. The mutual influence of electric dipole resonance between adjacent resonators decreases with the increase of parameter g. In Figure 5b, the relationship between the absorption spectrum and semi-major axis (a) of the resonator is shown, and other geometric parameters are fixed. As can be seen from the figure, the absorption band moves slightly to a longer wavelength having a larger parameter a, and the absorption efficiency also increases. Due to the increase of parameter a, the size of the resonator increases, and the light energy can be more effectively coupled to the surface of the structure. Figure 5c summarizes the evolution of the absorption spectrum when the semi-minor axis (b) of the resonator is changed while the other parameters are constant. As parameter b increases, the absorption spectrum gradually shifts red, but there is an obvious discrete absorption band in the spectrum, when the wavelength is about 650 nm, the absorption efficiency decreases with the increase of parameter b, which is due to the absence of the corresponding resonance state. Thus, with the change of parameters a, b in the absorber, high average absorption efficiency can be effectively maintained. At the same time, the perfect absorption window can be manipulated in the wavelength range.

**Figure 6:**
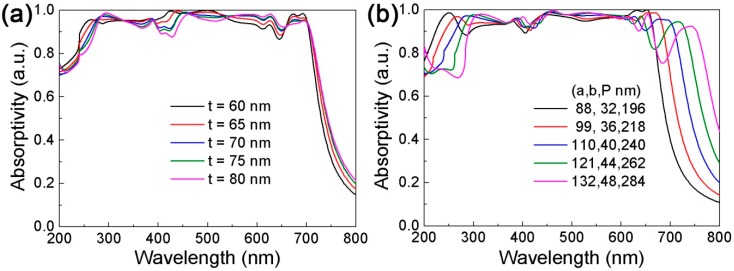
The influence of changing the thickness and period of the resonator in the wavelength range. (**a**) The absorption evolution of the resonator thickness from 60 nm to 80 nm; (**b**) in the absorption evolution under different periods, the ratio coefficient between the semi-major axis and the semi-minor axis is set as 2.75, which is consistent with the initial proportionality coefficient.

The thickness and period of the Si resonator also have an effect on the absorption spectrum. The spectral change of the proposed DBMPA when the parameter t of the resonator is changed is shown in Figure 6a. The parameter t increases gradually from 50 nm to 80 nm, the absorption spectrum redshifts continuously in the wavelength range, and the absorption efficiency increases in the short wavelength range. In the long wavelength range, the absorption efficiency decreases, the reason being that the dipole resonance excitation is the main absorption contribution in the short wavelength range, and the dipole resonance increases with the increase of parameter t. In the long wavelength range, the coupling effect of the dipole resonance and SPR is the main absorption contribution, which gets weaker. The absorption spectra with different structural parameters confirms the prediction of the high scalability of the proposed perfect absorber, is shown in Figure 6b. The ratio coefficient between the semi-major axis of the resonator and the semi-minor axis is selected to be 2.75, and parameters a, b and P are changed at the same time. As the above parameters increase, the absorption efficiency of the absorption spectrum produces small changes showing continuous redshift. When choosing the appropriate geometry, we can find the ideal case for perfect absorption. In the meantime, these features confirm the spectral tunability of the broad spectrum light absorber.

**Figure 7:**
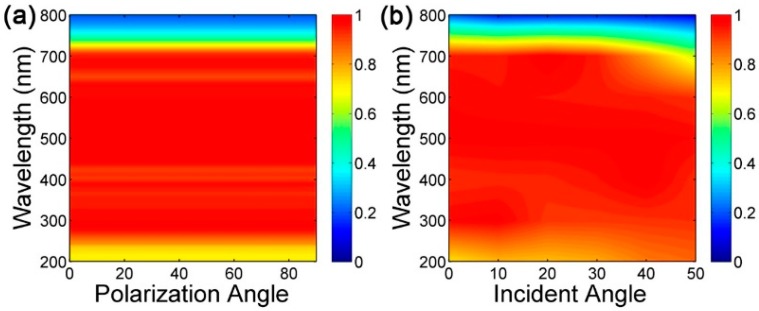
(**a**) The absorption evolution map when the polarization angle increases from 0 (TM, E parallel to *x*-axis) to 90 degrees (TE, E parallel to *y*-axis); (**b**) the absorption evolution map with the change of incident angle from 0 to 50 degrees with TM polarization state.

In addition to studying the effect of structural parameters on the absorption spectrum, we also studied the changes of the absorption spectrum under different polarization angles and incident angles, as shown in Figure 7a,b. As the polarization angle increases from 0 (TM, E parallel to *x*-axis) to 90 degrees (TE, E parallel to *y*-axis) in units of 10 degrees, the absorption evolution map shows a perfect absorption of a wide spectrum throughout the entire polarization angle, indicating that polarization-independent absorption is obtained, due mainly to the high symmetry [44,45] and local resonance of the structure. Polarization-independent DBMPA is widely used in optoelectronic devices including hot-electron equipment [46]. Figure 7b shows the absorption evolution with the change of incident angle with TM polarization state. When the incident angle is 50 degrees, the absorption spectrum is slightly blue-shifted in the long wavelength, and the perfect absorption in the wavelength range is maintained. It is confirmed that the incident angle is insensitive and can be used in a complex electromagnetic environment.

**Figure 8:**
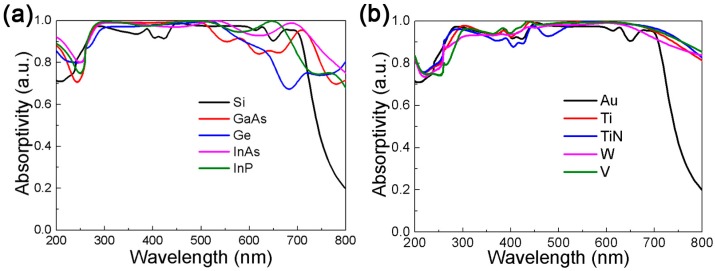
(**a**) The absorption spectrum when keeping the substrate unchanged and replacing Si with other dielectrics such as GaAs, Ge, InAs and InP. (**b**) The absorption spectrum when keeping the Si unchanged and replacing Au substrate with refractory materials such as Ti, TiN, W, V.

Finally, while keeping the size of the structure unchanged, we use different materials to replace the patterned Si and the Au substrate. The absorption response of the BMPAs is summarized in Figure 8. Figure 8a plots keeping the Au unchanged while replacing Si with other high-index dielectrics such as GaAs, Ge, InAs and InP. Its excellent properties enable InAs and InP to maintain higher absorption. Absorption evolution when the Si is fixed and the Au substrate is replaced with refractory materials is plotted in Figure 8b. The wide and flat absorption band of refractories is closely related to their own high intrinsic loss [47], of which, TiN and W compatible with CMOS [26]. The above results show that strong absorption can be observed in all the types of materials we have selected. Different materials are used in different fields, indicating that the proposed DBMPA will have a wide range of applications.

## 4. Conclusions

In summary, we have proposed and demonstrated a novel method for a subwavelength broadband metamaterial perfect absorber based on diamond dielectric arrays. The characteristics of an absorber composed of common materials are discussed in detail. In a subwavelength absorber with a thickness of 170 nm, considering the absorption rate of more than 90%, the spectral bandwidth of 447 nm is realized in the range of ultraviolet–visible light. The result of multipole decomposition of diamond resonator shows that the dipole resonance excitation and surface plasmon resonance are the main contributions to absorption. The wavelength range of the resonant absorption can be adjusted artificially to obtain a perfect tunable absorber. The proposed DBMPA achieves near-perfect polarization-independent and incident angle insensitive broadband light absorption. According to Kirchhoff’s law, the proposed absorber can also be used as heat radiator. In addition, perfect absorption is also achieved by using different materials instead of patterned Si and Au substrate, which will make the proposed DBMPA greatly broaden the application field.
